# The Impact of Stress on Women’s Sexuality in the First Months After Childbirth—A Pilot Cross-Sectional Comparative Study

**DOI:** 10.3390/jcm14030847

**Published:** 2025-01-27

**Authors:** Kornelia Zaręba, Maria Florkiewicz-Danel, Michał Ciebiera, Stanisław Wójtowicz, Yauhen Statsenko, Sara Maki, Jolanta Olszewska, Shamsa Al Awar, Grzegorz Jakiel

**Affiliations:** 1 Department of Obstetrics and Gynecology, College of Medicine and Health Sciences (CMHS), United Arab Emirates University (UAEU), Al Ain P.O. Box 15551, United Arab Emirates; sara.maki@uaeu.ac.ae (S.M.); sawar@uaeu.ac.ae (S.A.A.); 2 Department of Nursing, Faculty of Rehabilitation, Józef Piłsudski University of Physical Education in Warsaw, 00-968 Warsaw, Poland; mariaflorkiewicz@wp.pl; 3 2nd Department of Obstetrics and Gynecology, Center of Postgraduate Medical Education in Warsaw, 00-189 Warsaw, Poland; michal.ciebiera@gmail.com; 4 Warsaw Institute of Women’s Health in Warsaw, 00-189 Warsaw, Poland; 5 Department of Health Psychology, Medical University of Warsaw, 00-575 Warsaw, Poland; stwojt@o2.pl; 6 Old Polish University of Applied Sciences, 25-666 Kielce, Poland; 7 Department of Radiology, College of Medicine and Health Sciences (CMHS), United Arab Emirates University (UAEU), Al Ain P.O. Box 15551, United Arab Emirates; e.a.statsenko@uaeu.ac.ae; 8 Department of Obstetrics and Gynecology Nursing, Medical University of Gdańsk, 80-211 Gdańsk, Poland; jolanta.olszewska@gumed.edu.pl; 9 1st Department of Obstetrics and Gynecology, Center of Postgraduate Medical Education in Warsaw, 01-004 Waraw, Poland; grzegorz.jakiel1@o2.pl

**Keywords:** stress, anxiety, sexuality, maternity, puerperium, coping strategies

## Abstract

**Background:** The postpartum period can carry strong stress related to the sudden changes in a woman’s life, which may contribute to changes in the female sexual sphere. The aim of this study was to determine the impact of stress on women’s sexuality in the early motherhood period. **Methods:** A total of 111 women were studied, including 65 in the puerperal period and 46 women who constituted the control group. We used the author’s questionnaire and five standardized psychological questionnaires (CISS-21,STAI, PSS-10, SSS-W, and the Mell–Krat Scale for women). **Results:** Perceived stress (PSS-10 scores) was significantly lower (*p* < 0.001) and sexual satisfaction (SSS-W scores) was significantly higher both regarding the summary scores (*p* < 0.001) and in all subscales (contentment, communication, and compatibility). In the study group, the intensity of stress was negatively correlated with the level of sexual satisfaction (*p* = 0.014). Women with an emotional way of coping with stress (CISS-21 inventory) in both groups achieved higher scores of sexual satisfaction in terms of communication, but a lower level in terms of interpersonal contact (*p* = 0.007), but higher stress intensity scores in the PSS-10 scale and in both STAI questionnaire stems. **Conclusions:** The early period of motherhood does not increase stress levels or decrease sexual satisfaction. It is likely that the sense of stability promotes a reduction in stress levels, which contributes to better sexual satisfaction.

## 1. Introduction

The World Health Organization perceives sexual health as “fundamental to the overall health and well-being of individuals, couples and families” [1]. In the cycle of a woman’s life, we observe numerous changes in the sexual sphere, resulting from factors like changes in social roles, which undoubtedly include the period of pregnancy and puerperium [2]. In this sensitive period, in addition to the obvious changes in the social sphere, biological changes also occur, such as hormonal or emotional ones, including stress related to the need to constantly care for a child [3]. Additional factors affecting sexual function include personality traits of the individual and general health, which are subject to further changes in stressful situations [4]. The course of pregnancy and childbirth or any traumatic experiences associated with them are also of importance [5,6].

We define sexual satisfaction as positive sexual experiences, which are the foundation for maintaining a relationship and strengthening the partnership bond [7,8]. Lawrence and Byers defined sexual satisfaction as “an affective response arising from a subjective assessment of a sexual relationship” [9]. The determinants of sexual response include both individual and relational aspects [10]. The sexual satisfaction rating scale proposed in 2005 by Meston and Trapnell, and used in this study, was developed to evaluate the relational aspect in the dimensions of communication, compatibility, and contentment [11].

The concept of stress in the postpartum period attracts social interest due to numerous complications both for the mother and the whole family [12]. Foklman and Lazarus defined stress as the negative well-being of an individual in relation to the environment [13]. Undoubtedly, the period of motherhood is a strong stressor in all areas of a woman’s life including sleep pattern, mental stress, relationship issues, blood pressure, and general well-being [14]. Additionally, maternal stress may affect the child’s behavior and its future development, leading to growth disorders, poor language and cognitive development, sleep problems, and even Polycystic Ovary Syndrome [15,16].

The inability to cope with stress is the major cause of complications in both the mother and the child, leading to cognitive deficits and disorders in the child and depressive disorders in the mother [12,17,18]. It was confirmed that mothers who used such strategies as acceptance and positive reframing coped significantly better with stress [12]. The ability to cope with stress depends on numerous factors, both behavioral and psychological [19]. The body develops autonomic mechanisms to maintain homeostasis in response to stress. However, the excessive activity of physiological processes may lead to health complications [20].

Available studies concerning the importance of stress in women’s sexuality yielded inconclusive results [21,22]. Bodenmann et al. demonstrated that stress, depending on its intensity and nature, might lead to sexual disorders in a given relationship [22]. Bivariate correlations revealed that higher levels of marital satisfaction correlate with lower levels of internal daily stress (r = −0.35 for men; r = −0.45), sexual satisfaction (r= −0.049), and sexual activity (−0.45), and with higher levels of sexual dysfunction for men and for women. Burii and Carvalheira suggested a stimulating effect of stress on the incidence of sexual activity. Sexual activity is believed to be a strategy for coping with stress and may be viewed as a relaxation method (22.6%) [23]. However, few authors described the influence of stress on female sexuality in the postpartum period [2,24]. Laumann et al. described a distinct correlation between broadly understood emotional disorders and stress and sexual dysfunctions in postpartum women [24]. Tavares noted a relationship between positive sexual experiences and a lower perception of stress in couples in the early days of motherhood [2].

Due to the inconclusive results of studies describing women’s sexuality after childbirth and in search of its determinants, we decided to conduct our own study among healthy women in early motherhood (6–8 weeks after childbirth), in whom no other risk factors for sexual dysfunction were identified.

## 2. Materials and Methods

The presented study is part of a project called “The impact of stress, fatigue, and emotions on the level of sexual needs during early motherhood”. This study was conducted in accordance with the Declaration of Helsinki and was approved by the Bioethics Committee of the Center of Postgraduate Medical Education (CPME) in Warsaw (approval number 72/PB/2018).

The study group comprised patients who gave birth at the Żelazna Medical Center Ltd. in the local birth center, where the 1st Department of Obstetrics and Gynecology of the CPME is located. In this phase of the project, the study group included patients staying in the Obstetrics Department. The patients had spontaneous vaginal deliveries without complications. They were randomly selected and recruited on the second day after labor. Subsequently, participants were instructed to fill out an anonymous questionnaire approximately 6 to 8 weeks following childbirth. During this time, patients usually come for a scheduled postnatal visit. The control group included women of reproductive age recruited using information in the social media of outpatient clinics.

A total of 111 women who met the specific inclusion criteria were examined.

The criteria for inclusion in the study group were as follows:

- Age range: 18 to 45 years;
- Spontaneous pregnancy (after exclusion of assisted reproductive techniques);
- Absence of risk factors for preterm birth;
- Absence of any significant pregnancy-related pathology;
- No history of severe chronic diseases or medical conditions with potential contributions to the study outcomes (e.g., neoplastic diseases);
- The second day of puerperium after a vaginal delivery;
- Informed consent to participate in the study;
- Vaginal delivery.

The criteria for exclusion from the study group were as follows:

- Cesarean section;
- Presence of significant medical complications during the puerperium, such as hemorrhage, severe inflammation, or a body temperature exceeding 38 °C;
- Hospitalization for reasons other than physiological stay after delivery during the postpartum period;
- Using hormonal contraception.

A total of 82 questionnaires were distributed in the study group, and 65 questionnaires were returned. The return rate of questionnaires was 79.3% in the group. Inclusion and exclusion criteria comprised healthy patients without any additional risk factors that can influence the risk of stress and sexual dysfunctions. Specifically, we aimed to include women without risk factors for sexual dysfunctions especially connected with early motherhood (nulliparous women or women with children over 6 years of age).

The control group included nulliparous women or those whose children were over 6 years of age.

The inclusion criteria for the control group were as follows:

- Age range: 18 to 45 years;
- Nulliparous women or women with children over 6 years of age;
- No severe chronic diseases or relevant medical history, including neoplastic conditions;
- No use of contraception during the study;
- No endocrine diseases that could affect survey results;
- No psychiatric disorders that could affect survey results;
- Informed consent to participate in the study.

The exclusion criteria for the control group were as follows:

- Refusal to provide consent to participate in the study;
- Disclosure of relevant medical events between the receipt and return of the survey.

In the control group, 60 surveys were distributed and 46 were returned, which accounted for a 76.6% survey return rate.

Questionnaires

This study used a survey developed for its purposes, as well as five surveys standardized and accredited by the Polish Psychological Society and the Polish Sexological Society: S. Cohen, T. Camarck, R. Mermelstein—the Perceived Stress Scale (PSS-10), the Sexual Satisfaction Scale for Women (SSS-W), the Mell–Krat Scale for women, the STAI questionnaire, and the CISS-21 questionnaire. A total of 111 women who met the specific inclusion criteria were examined.

The present authors’ questionnaire

This study employed a questionnaire comprising five sections: sociodemographic data, medical history, and gynecological and obstetric history including sexual history. Sociodemographic information was collected by inquiring about participants’ age, education, place of residence, religion, marital status, and financial situation.

The Sexual Satisfaction Scale for Women (SSS-W)

The SSS-W scale, developed by Meston and Trapnell in the United States, is a tool designed to assess sexual satisfaction and distress in women [11]. An adapted version of the scale was created in Poland by K. Janowski, W. Karłowicz, and M. Kubiak. The scale consists of 18 items, which are organized into three subscales: contentment, communication, and compatibility. The authors defined contentment as a comprehensive assessment of satisfaction with sexual functioning, including closeness, frequency of sexual contact, and intimacy. The communication dimension determines the ability of partners to communicate freely in terms of sexual matters and the ability to communicate with each other in relation to the deepest feelings and emotions. Compatibility refers to the degree of similarity between partners in attitudes, needs, beliefs, desires, and preferences related to sex, as well as the level of sexual attraction felt towards one another. Responses are provided on a 5-point Likert scale, with subscale scores ranging from 6 to 30, and a total score ranging from 18 to 90 for the entire survey. The reliability coefficient (Cronbach’s α) for the overall scale was found to be high at 0.93 [25].

The Mell–Krat questionnaire

The Mell–Krat questionnaire, developed by Mell and Kratochvil, is designed to assess individuals’ sexual needs and responses [26]. The version adapted by Kromierzynska comprises 20 items and is a modification of the original tool. This scale evaluates various aspects related to sexual reactivity, including libido, arousal, and the frequency of orgasm [27]. In the Mell–Krat questionnaire, each item is scored on a scale from 1 to 4 points. The maximal possible score is 80. The cut-off point for low sexual reactivity is below 55 points. The reliability coefficient (Cronbach’s α) for the scale is 0.69 [28].

PSS-10 (Perceived Stress Scale-10)

The Perceived Stress Scale (PSS) was developed by Cohen, Kamarck, and Mermelstein in 1983 and adapted in Poland by Juczyński and Ogińska-Bulik in 2009 [29]. PSS is a self-report tool that assesses the intensity of stress related to a life situation. It evaluates thoughts and feelings that have taken place over the past month on a scale from 0 “never” to 4 “very often”. The results of the scale show the general psychological comfort associated with coping with problems that occur. PSS scores are obtained by reversing responses (e.g., 0 = 4, 1 = 3, 2 = 2, 3 = 1, and 4 = 0) to four positively stated items (items 4, 5, 7, and 8) and then summing across all scale items. The scale has marked psychometric properties. According to a review manuscript on 12 studies, the Cronbach’s alpha of the PSS-10 was >0.70 in all studies [30].

STAI (State–Trait Anxiety Inventory)

The State–Trait Anxiety Inventory (STAI) consists of two components that assess state anxiety and trait anxiety. Each item consists of four answers. Consequently, respondents can score between 20 and 80 points for each component, with higher scores indicating a higher level of anxiety. The results for both state and trait anxiety are further categorized into sten scores (ranging from 1 to 10).

The sten scores for both parts are divided into three levels:

Sten scores from 1 to 3: Low state anxiety and trait anxiety;

Sten scores from 4 to 7: Moderate state anxiety and trait anxiety;

Sten scores from 8 to 10: High state anxiety and trait anxiety.

The tool has a good internal consistency, reaching Cronbach’s α = 0.89 [31].

CISS-21 (Coping Inventory for Stressful Situations)

The CISS-21 is a four-factor model of coping with difficult and stressful situations [32]. It is a 48-item self-report inventory designed to assess stress coping strategies across three dimensions: task-oriented coping (TOS), emotion-oriented coping (EOS), and avoidance-oriented coping (AOS). Each dimension consists of 16 items. Respondents indicate the frequency of their reactions by selecting the appropriate number on a five-point scale (1 = never, 2 = very rarely, 3 = sometimes, 4 = often, 5 = very often). The Cronbach’s alpha reliability coefficients for the subscales are as follows: 0.83 for TOS and EOS, 0.69 for AOS, 0.65 for DS, and 0.57 for SDS [33].

Statistics

The data were analyzed using IBM SPSS Statistics version 28 (New York, 2021), with a significance level set at *p* < 0.05. The Shapiro–Wilk test was applied to assess the normality of the data distribution. Descriptive statistics, including frequencies (both numeral and percentage), means, and standard deviations, were calculated. For quantitative data that did not meet the assumption of normality, non-parametric tests were employed. The Mann–Whitney U test was used for comparisons between two groups, while the Kruskal–Wallis test was applied for comparisons involving more than two groups. The Bonferroni test was used as a post-hoc procedure. Given the use of an ordinal scale, the Kendall tau-b correlation coefficient was applied for statistical analysis. Additionally, the frequency of intercourse was converted into an ordinal scale (ranging from 1 = several times a year to 7 = several times a day).

## 3. Results

Regarding age, the largest group of respondents included women aged 28–30 (over 26%) in the study group, and women under 25 years of age (19.6%) in the control group (Table 1). The mean age in the study group was 31.57 (SD 3.97), and in the control group it was 31.65 (SD 6.88). The majority of the partners in the study group were men aged 31–33 years (24.6%), and the mean age was 34.62 (SD 5.46). Similarly, individuals under 25 years of age (19.6%) constituted the largest group of men in the control group. The average age was 31.77 (SD 10.49). In both groups, the majority of respondents lived in urban areas: 83.2% in the study group and 87% in the control group. Over 80% of women in the study group and 63% of women in the control group had higher education. Most of the respondents in the study group had partners who had higher education (72.3%). In the control group, men with higher education also constituted the largest group (43.5%). Significantly more single (never married) women were in the control group (56.5%) compared to the study group (21.5%). The majority of study group women (80%) worked during pregnancy. The respective percentage in the control group was 79%. However, this group included a higher percentage of students. Women from the study group whose parents had a secondary education accounted for 52.3%. Regarding the control group, it was 47.8% of the respondents. Only 1.5% of the women in the study group and 4.3% in the control group reported financial problems.

Women in the study group reported intercourse more often (55.4% reported intercourse at least twice a week) than those in the control group (30.5%, at least twice a week). No significant intergroup difference was noted in terms of sexual activity types.

The chi-squared test analysis of both groups revealed statistically significant differences in marital status (*p* = 0.001), frequency of intercourse (*p* = 0.008), and number of children (*p* = 0.0001). There was no statistical difference in the types of sexual activities for oral (*p* = 0.656), anal (*p* = 0.449), and vaginal (*p* = 1.0) sexual intercourse.

Only 17.1% of respondents reported high stress levels, while 40.2% experienced average stress levels, and 28.1% reported low stress levels. This study found no significant impact of demographic variables on perceived sexual satisfaction or stress levels in either the study or control group.

The study group exhibited significantly lower perceived stress levels (PSS-10 scores) and significantly higher sexual satisfaction (SSS-W scores) compared to the control group, particularly in the subscales of contentment, communication, and compatibility (Table 2 and Table 3).

The assessment of sexual satisfaction using the Mell–Krat questionnaire showed no significant differences between the groups (Table 3).

In the study group, the intensity of stress was negatively correlated with sexual satisfaction, as measured by both the Mell–Krat questionnaire and the PSS-10. In contrast, no significant correlations were observed between the SSS-W and PSS-10 scores within the study group, nor between the PSS-10 stress levels and sexual satisfaction (as assessed by both the SSS-W and Mell–Krat questionnaires) in the control group (Table 4).

However, the Kruskal–Wallis test showed a significant difference in the frequency of intercourse between people with different levels of stress (*p* = 0.030). Linear dependence analysis was performed using Kendall’s tau-b correlation coefficient. However, no significant correlation was shown between stress level and the frequency of intercourse (r = 0.086, *p* = 0.382).

The Kendall tau-b correlation performed in the study group revealed that the level of sexual satisfaction (the aspect of communication) was positively correlated with the emotional style of coping with stress (CISS-21 inventory) (*p* = 0.025) (Table 5). A similar relationship was observed in the control group (*p* = 0.070 with the significance level of *p* = 0.010). Conversely, we noted a negative correlation with the level of sexual satisfaction and the compatibility (*p* = 0.028). The level of sexual satisfaction in the aspect of communication was also negatively correlated with the search for interpersonal contact (*p* = 0.010). A similar phenomenon was noted in the control group (*p* = 0.007).

Women from the study group characterized by an emotional style of coping with stress had higher scores of perceived stress according to the PSS-10 scale (*p* = 0.28 with a significance level of *p* = 0.05). A similar phenomenon was noted in the control group (*p* = 0.02).

Statistically significant correlations were also observed in terms of coping with stress regarding stress and anxiety intensity in the study group (Table 6). A positive correlation was noted in both stens (STAI_X1 *p* = 0.031, STAI_X2 *p* = 0.000) in women characterized by an emotional nature. Similar results were obtained by respondents characterized by avoidant coping with stress (STAI_X1 *p* = 0.034 and STAI_X2 *p* = 0.001). In the control group, similar results were only found in the second sten (STAI_X2), i.e., identical results were obtained for the emotional and task-oriented ways of coping with stress (*p* = 0.000). In addition, study group women with a high level of state anxiety more often sought interpersonal contact (*p* = 0.004). Respondents with a high level of trait anxiety more often coped with stress by means of distraction (*p* = 0.001). Such outcomes were not observed in the control group.

## 4. Discussion

This research presents comprehensive aspects of women’s sexuality after childbirth and modes of efficient coping strategies in this important field of a female’s life. It highlights that the level of stress was lower in women during early puerperium and the level of sexual satisfaction (SSS-W scores) was higher, especially regarding the subscales of contentment, communication, and compatibility. In the study group, the intensity of stress was negatively correlated with sexual satisfaction and had a significant impact on the frequency of intercourse in the study group. Women in both groups characterized by an emotional style of coping with stress (CISS-10) achieved higher stress intensity scores in the PSS-10 scale and higher scores of stress and anxiety in STAI questionnaire stens. Moreover, this group reported a high level of sexual satisfaction (communication domain) and a low level in the interpersonal contact domain (*p* = 0.007).

Stress occurring in the postpartum period may have both stimulating and inhibiting effects on women’s sexuality [24]. The situation might be influenced by numerous factors, such as the timing of the study, the age of the participants and partners, marital status, or the stability of the life and partner situation. The study group and the control group differed significantly in terms of marital status and the level of education of the partner. These variables may have influenced different perceptions of stress and sexual satisfaction in the groups. The younger age and higher number of students in the control group might be associated with less sexual experience in the group and a lower sense of life stability. This may also explain the more frequent sexual activity of women in the study group compared to the control group [34]. Conversely, students have more freedom to make new sexual contact. In the case of women, frequent partner changes or relationship instability may affect their sexuality, and even lead to mood disorders [35,36,37]. Due to the female biological cycle, it is difficult to recruit a control group to match the age of women during early puerperium. Therefore, such a group often includes students matching the study group in terms of the educational profile. Similarly, Chaaya et al. included 97 women in the postpartum period (6 months after childbirth) in the study group, and a group of healthy female students (*n* = 58) in the control group [38]. University students were significantly younger, better educated, single, and scored higher on stress levels compared to women who had delivered [38]. This was probably due to the stress associated with exams and the uncertainty regarding future work and family situation. In the present study, the study group differed from the control group in terms of marital status and age of the partner, which may have influenced differences in the perceived level of stress in the groups and the level of sexual satisfaction related to, for example, its occasional nature [39]. In Poland, students often live with their parents or in dormitories. Occasionally, they live with their partners. Therefore, sexual contact may be less frequent and irregular. They often work apart from studying, which makes it difficult to have regular sexual contact [40].

The results regarding the sexuality of mothers in early motherhood are ambiguous in the available literature. Research by Ahlborg et al. showed that, despite happiness, the period of parenthood was associated with a decrease in sexual function in the relationship [20]. Moreover, a study by Mirzeai et al. revealed that non-pregnant and non-breastfeeding women had a much lower degree of sexual dysfunction in terms of desire, arousal, orgasm, and pain compared to nursing mothers [41]. Similarly, research conducted by Zgliczyńska et al. in a group of 433 women comparing sexuality before pregnancy and after childbirth showed that childbirth and motherhood had a negative impact on sexuality [42]. However, the present study did not confirm those data. This may be due to the fact that different questionnaires to assess women’s sexuality were used, the study was conducted relatively shortly after childbirth, and the study by Zgliczyńska et al. included a more diverse population of women in terms of obstetric history, which can increase the risks of sexual disfunctions.

Regarding the timing of research, the study by Woolhouse et al. conducted on a group of 1507 women examined at 3, 6, and 12 months and 4.5 years after childbirth revealed that significant improvement in sexual activity was noted at 12 months after delivery [43]. However, Connolly et al. conducted a study in women at 6, 12, and 24 weeks postpartum showing that, 6 weeks after delivery, most women no longer reported pain related to vaginal intercourse [44]. Most probably, the reason for late sexual activity was not connected with the biological aspects of sexuality. Conversely, research by Matthies showed that sexual function decreased from the third trimester to the short postpartum period (1 week), and then increased to its prepartum state, however, at 4 months after delivery [45]. Similarly, Hipp et al., reported that, in the first 6 weeks after birth, vaginal intercourse was the preferred form of sexual activity in only 26% of the respondents [46]. Perhaps the decrease in sexual satisfaction was due to the stress associated with motherhood. The present study did not show similar results, as we did not observe such a phenomenon.

The present study showed that women during early puerperium had lower levels of perceived stress. A study conducted by Leavitt et al. in a group of 169 couples showed that increased stress levels occurring 6 months after childbirth had a negative effect on sexual satisfaction even 12 months after childbirth. Moreover, stress was associated with a reduced satisfaction with sex life in women [22]. Conversely, Hipp et al. conducted a study on 304 women and showed that stress had no significant effect on women’s sexuality [46]. Notably, the present study group included women who had delivered in a birth center, which means that the women preferred the most natural forms of childbirth, which can decrease the level of perceived stress connected with the medicalization of delivery and hospitalization.

Research by McDonald et al. showed that emotional satisfaction with intimate partner relationships tended to decrease from 67.3% of the respondents declaring high satisfaction after 3 months to 53.9% after 18 months after childbirth. There was a strong association between emotional satisfaction and the degree to which women experienced physical pleasure during sexual activity [47]. This may be due to the fact that the level of stress during early puerperium is reduced and the level of tiredness is increased [48]. In the present study, women in the study and control groups characterized by an emotional style of coping with stress had a higher level of stress on the PSS-10 scale in both stens of the STAI questionnaire. Interestingly, both in the study group and in the control group, women with an emotional way of coping with stress (CISS-21 inventory) achieved a higher level of sexual satisfaction in the aspect of communication, but a lower level in terms of interpersonal contact. Therefore, such a relationship may result from the personality of the surveyed women rather than from the life situation itself. It is known that the pattern of experiencing one’s own sexuality among women is extremely complex and changes during the relationship, being additionally dependent on the life situation of the respondents [49]. According to the concept of the sexual response cycle by Basson, women attract more attention to non-sexual gratification such as emotional intimacy, which is the main motive and purpose of engaging in sexual activity in women [50,51]. This concept applies especially to people who are in stable relationships. Sexual satisfaction itself is, therefore, understood as an increase in commitment and intimacy in a relationship, including such aspects as trust, a sense of closeness, respect, and communication [50,51]. Therefore, this may explain different levels of sexual satisfaction depending on the type of personality and the way of coping with stress. Similarly, Basson’s theory may explain lower sexual satisfaction in women with higher levels of stress assessed with the Mell–Krat questionnaire, which mostly covers sexual dysfunctions, that was not confirmed with the SSS-W questionnaire, which covers the relational and psychological, rather than biological, aspects of sexuality. In the present study, the level of sexual satisfaction (SSS-W scores) was significantly higher in the study group in the subscales of contentment, communication, and compatibility. Moreover, the majority of sexology studies are based on the Female Sexual Function Index (FSFI), which was not used in this study. Therefore, some of the cited results may differ in their results.

When examining factors that influence women’s sexuality, it is essential to consider the hormonal fluctuations that occur during the postpartum period. During this time, there is a decrease in estrogen and progesterone levels, while prolactin levels rise in breastfeeding mothers [52,53]. Additionally, changes in cortisol, oxytocin, and androgens, such as DHEAS, may also play a role in altering sexual function, often contributing to a reduction in sexual desire and activity [53]. These hormonal shifts must be factored into discussions about potential sexual dysfunctions in early motherhood and may be correlated with stress and anxiety [53]. Consequently, the second part of this project focused on assessing the hormonal balance of young mothers and its impact on their sexuality, as well as the role of stress in influencing these hormonal changes. The findings will be presented in subsequent manuscripts.

### 4.1. Limitations

The present study has numerous limitations. It is a cross-sectional study based on self-assessment surveys, which present a subjective assessment of the situation. Standardized psychological tests were used to make the results more objective. However, the surveys included an explicit question that could increase the rate of false positive results. Another limitation is related to the single-center nature of the study and slight differences in the size of the control group compared to the study group. The sample size is small, including only 65 women in the postpartum group, which may not be sufficient to draw scientifically reliable conclusions. Due to the comprehensive nature of the project (additional hormonal assessment requiring tests performed during the first phase of the menstrual cycle), and the relocation of the principal investigators, the study was conducted in a small group of women, which is not representative for the entire population. A larger and more diverse sample is planned to provide more robust results. In addition, the study and control groups included relatively young women, who cannot constitute a representative group for the whole country. Because of the aim to include women without risk factors for sexual dysfunctions especially connected with early motherhood (nulliparous women or women with children over 6 years of age), the control group comprised younger females who were mostly unmarried and, because of the age, their partners had lower partner education. Therefore, the present study was a pilot one, and we plan to repeat it in a larger group of patients as a longitudinal study. A longitudinal study would provide a clearer picture of how stress and sexual satisfaction evolve over time during the postpartum period. Longitudinal data could also help assess causality more effectively.

### 4.2. Strengths

This is a pioneering study conducted in healthy women during puerperium following a physiological delivery. The study group included healthy women after childbirth, recruited in the birth center of a maternity hospital, which guaranteed the physiological course of pregnancy and childbirth. Thus, perinatal and peripartal risk factors for sexual disorders, such as anxiety related to pregnancy and childbirth, were eliminated. To strengthen the effect, young healthy women were recruited as the control group. Moreover, numerous validated psychological scales were used in this study, which further strengthens the importance of the obtained results. The use of the CISS-21 questionnaire made it possible to determine optimal ways of coping with stress in order to improve sexual satisfaction, which set the direction for subsequent psychological interventions in patients with postpartum sexual dysfunctions.

## 5. Conclusions

The ability to manage stress properly during the early period of motherhood may have a positive effect on the level of sexual satisfaction. It is likely that the sense of stability promotes a reduction in stress levels, which contributes to an increase in the level of sexual satisfaction. In view of the results, a task-oriented approach to stress and motherhood seems to be the best direction for psychological support and intervention.

## Figures and Tables

**Table 1:** Sociodemographic data of the respondents in the study group (*n* = 65) and control group (*n* = 46).

Variable	Study Group *n* (%)	Control Group *n* (%)
**Level of education—partner**		
Master’s degree	47 (72.3%)	20 (43.5%)
Bachelor’s degree	3 (4.6%)	9 (19.5%)
Secondary	15 (23.1%)	12 (26.1%)
Vocational	0 (0.0%)	3 (6.5%)
Primary	0 (0.0%)	1 (2.2%)
No data	0 (0.0%)	1 (2.2%)
**Level of education—parents**		
Master’s degree	24 (36.9%)	16 (34.8%)
Bachelor’s degree	6 (9.2%)	9 (19.5%)
Secondary	28 (43.1%)	19 (41.3%)
Vocational	0 (0.0%)	1 (2.2%)
Primary	7 (10.8%)	1 (2.2%)
**Age**		
Under 25	1 (1.5%)	12 (26.1%)
25–30	26 (40.0%)	8 (17.3%)
30–35	26 (40.0%)	9 (19.6%)
35–40	11 (16.9%)	10 (21.8%)
40+	1 (1.5%)	7 (15.2%)
**Age of the partner**		
Under 25	1 (1.5%)	9 (19.6%)
25–30	12 (18.5%)	10 (21.7%)
30–35	27(41.5%)	7 (15.2%)
35–40	13 (20%)	5 (10.9%)
40+	12 (18.5%)	11 (23.9%)
No data	0 (0.0%)	4 (8.7%)
**Place of residence**		
Village	8 (12.3%)	6 (13.0%)
Province capital	45 (69.2%)	36 (78.3%)
District capital	5 (7.7%)	3 (6.5%)
Other towns	4 (6.2%)	1 (2.2%)
No data	3 (4.6%)	0 (0.0%)
**Marital status**		
Single (never married) woman	14 (21.5%)	26 (56.5%)
Married	50 (76.9%)	15 (32.6%)
Divorced	1 (1.5%)	3 (6.5%)
Widow	0 (0.0%)	1 (2.2%)
No data	0 (0.0%)	1 (2.2%)
**Professional work**		
Does not work professionally	12 (18.5%)	9 (19.5%)
Works professionally	52 (80.0%)	36 (78.3%)
No data	1 (1.5%)	1 (2.2%)
**Financial status**		
A lot of money	2 (3.1%)	0 (0%)
Enough money	27 (41.5%)	10 (21.7%)
Average level	34 (52.3%)	34 (73.9%)
Insufficient amount of money	1 (1.5%)	2 (4.3%)
No data	1 (1.5%)	0 (0%)
**Frequency of intercourse**		
Once a month or less frequently	4 (6.1%)	4 (8.8%)
Several times a month or once a week	25 (38.5%)	23 (49.9%)
Twice a week or more	36 (55.4%)	14 (30.5%)
No data	0 (0.0%)	5 (10.8%)
**Type of sexual activity (multiple answers possible)**		
Oral sex	44 (67.7%)	30 (66.6%)
Anal sex	13 (20.0%)	12 (26.4%)
Vaginal sex	65 (100%)	46 (100%)
**Number of children**		
0	0 (0.0%)	34 (73.9%)
1	32 (49.2%)	5 (10.8%)
2	25 (38.5%)	6 (13.1%)
3 or more	8 (12.3%)	1 (2.2%)

**Table 2:** Comparison of perceived stress levels (PSS-10) between the study and control groups.

	Group	N	Mean	Median	SD	Min	Max	The Mann–Whitney U Test	
								Statistics	*p*-Value
Total PSS-10	study	65	17.05	18.00	6.596	4	31	5.091	<0.001
	control	46	23.61	23.00	4.166	16	34		

**Table 3:** SSS-W, Mell–Krat results in the groups.

	Group	N	Mean	Median	SD	Min	Max	The Mann–Whitney U Test	
								Statistics	*p*-Value
SSS-W Contentment	study	65	20.74	22.00	4.738	10	27	−5.659	<0.001
	control	46	15.02	14.00	3.700	10	22		
SSS-W Communication	study	65	22.77	23.00	5.049	10	30	−7.852	<0.001
	control	46	11.86	11.00	3.901	6	22		
SSS-W Compatibility	study	65	24.22	25.00	4.872	12	30	−8.195	<0.001
	control	46	11.35	10.00	4.180	6	21		
Total SSS-W	study	65	67.72	69.00	12.140	40	86	−8.099	<0.001
	control	46	38.23	37.0	10.172	22	61		
Total Mell–Krat	study	65	52.05	52.00	8.847	29	72	0.687	0.492
	control	46	53.19	55.00	9.570	25	76		
Mell–Krat deviation from the optimal level	study	65	−2.95	−3.00	8.847	−26	17	0.687	0.492
	control	46	−1.81	0.00	9.570	−30	21		

**Table 4:** Correlation between stress levels and sexual satisfaction in the study and control groups.

Correlations			
		Total PSS-10	
		Study Group	Control Group
SSS-W Contentment	r_s_	−0.228	0.118
	*p*	0.068	0.450
	N	65	43
SSS-W Communication	r_s_	−0.157	0.198
	*p*	0.213	0.203
	N	65	43
SSS-W Compatibility	r_s_	−0.226	0.165
	*p*	0.071	0.292
	N	65	43
Total SSS-W	r_s_	−0.221	0.180
	*p*	0.077	0.240
	N	65	43
Total Mell–Krat	r_s_	−0.307 *	0.203
	p	0.014	0.196
	N	63	42

r_s_—correlation value; N—number of data points. * Correlation is significant at the level of 0.05 (two-tailed).

**Table 5:** Correlation between coping with stress (CISS-21) and sexual satisfaction (SSS-W).

		Study Group				Control Group			
CISS-21		Contentment	Communication	Compatibility	SSS-W	Contentment	Communication	Compatibility	SSS-W
Task-oriented style	r	−0.091	−0.124	0.097	−0.049	−0.044	−0.482 **	0.237	−0.151
	p	0.319	0.161	0.274	0.581	0.775	0.001	0.126	0.333
	N	65	65	65	65	44	43	43	43
Emotional style	r	0.122	0.198 *	−0.223 *	0.003	0.034	0.408 **	−0.225	0.103
	*p*	0.182	0.025	0.012	0.968	0.827	0.007	0.146	0.513
	N	65	65	65	65	44	43	43	43
Avoidance style	r	−0.036	−0.128	0.028	−0.046	−0.104	−0.056	0.128	0.041
	*p*	0.695	0.151	0.750	0.604	0.500	0.719	0.412	0.794
	N	65	65	65	65	44	43	43	43
Distraction	r	0.090	−0.030	−0.100	−0.044	0.104	0.287	0.037	0.297
	*p*	0.327	0.737	0.264	0.616	0.502	0.062	0.813	0.053
	N	65	65	65	65	44	43	43	43
Searching for interpersonal contact	r	−0.218 *	−0.232 *	0.128	−0.122	−0.106	−0.403 **	0.226	−0.126
	*p*	0.019	0.010	0.157	0.175	0.494	0.007	0.144	0.422
	N	65	65	65	65	44	43	43	43

r—correlation value; N—number of data points. * Correlation is significant at the level of 0.05 (two-tailed). ** Correlation is significant at the level of 0.01 (two-tailed).

**Table 6:** Correlation between stress coping strategies (CISS-21) and levels of perceived stress and anxiety (STAI).

CISS-21		Study Group		Control Group	
		STAI_X1	STAI_X2	STAI_X1	STAI_X2
Task-oriented style	r	0.013	−0.107	−0.079	−0.543 **
	*p*	0.882	0.227	0.609	0.000
	N	65	64	44	44
Emotional style	r	0.190 *	0.492 **	0.238	0.678 **
	*p*	0.031	0.000	0.120	0.000
	N	65	64	44	44
Avoidance style	r	0.188 *	0.291 **	0.276	0.008
	*p*	0.034	0.001	0.069	0.958
	N	65	64	44	44
Distraction	r	0.134	0.302 **	0.073	0.208
	*p*	0.133	0.001	0.638	0.176
	N	65	64	44	44
Searching for interpersonal contact	r	0.256 **	0.091	0.208	−0.280
	*p*	0.004	0.313	0.175	0.065
	N	65	64	44	44

r—correlation value; N—number of data points. * Correlation is significant at the level of 0.05 (two-tailed). ** Correlation is significant at the level of 0.01 (two-tailed).

## Data Availability

The original contributions of this study are included in the article; further inquiries can be directed to the corresponding author.
